# Surveillance of Infection Severity: A Registry Study of Laboratory Diagnosed *Clostridium difficile*

**DOI:** 10.1371/journal.pmed.1001279

**Published:** 2012-07-31

**Authors:** Iryna Schlackow, A. Sarah Walker, Kate Dingle, David Griffiths, Sarah Oakley, John Finney, Ali Vaughan, Martin J. Gill, Derrick W. Crook, Tim E. A. Peto, David H. Wyllie

**Affiliations:** 1 NIHR Oxford Biomedical Research Centre, John Radcliffe Hospital, Oxford, United Kingdom; 2 MRC Clinical Trials Unit, London, United Kingdom; 3 Health Protection Agency, Oxford, United Kingdom; 4 University Hospitals Birmingham NHS Foundation Trust, United Kingdom; Brown University School of Medicine, United States of America

## Abstract

**Background:**

Changing clinical impact, as virulent clones replace less virulent ones, is a feature of many pathogenic bacterial species and can be difficult to detect. Consequently, innovative techniques monitoring infection severity are of potential clinical value.

**Methods and Findings:**

We studied 5,551 toxin-positive and 20,098 persistently toxin-negative patients tested for *Clostridium difficile* infection between February 1998 and July 2009 in a group of hospitals based in Oxford, UK, and investigated 28-day mortality and biomarkers of inflammation (blood neutrophil count, urea, and creatinine concentrations) collected at diagnosis using iterative sequential regression (ISR), a novel joinpoint-based regression technique suitable for serial monitoring of continuous or dichotomous outcomes. Among *C. difficile* toxin-positive patients in the Oxford hospitals, mean neutrophil counts on diagnosis increased from 2003, peaked in 2006–2007, and then declined; 28-day mortality increased from early 2006, peaked in late 2006–2007, and then declined. Molecular typing confirmed these changes were likely due to the ingress of the globally distributed severe *C. difficile* strain, ST1. We assessed the generalizability of ISR-based severity monitoring in three ways. First, we assessed and found strong (*p*<0.0001) associations between isolation of the ST1 severe strain and higher neutrophil counts at diagnosis in two unrelated large multi-centre studies, suggesting the technique described might be useful elsewhere. Second, we assessed and found similar trends in a second group of hospitals in Birmingham, UK, from which 5,399 cases were analysed. Third, we used simulation to assess the performance of this surveillance system given the ingress of future severe strains under a variety of assumptions. ISR-based severity monitoring allowed the detection of the severity change years earlier than mortality monitoring.

**Conclusions:**

Automated electronic systems providing early warning of the changing severity of infectious conditions can be established using routinely collected laboratory hospital data. In the settings studied here these systems have higher performance than those monitoring mortality, at least in *C. difficile* infection. Such systems could have wider applicability for monitoring infections presenting in hospital.

## Abstract 2

Iryna Schlackow and colleagues investigated whether electronic systems providing early warning of changing severity of infectious conditions can be established using routinely collected laboratory hospital data. They showed that for *Clostridium difficile* infection, these systems perform better than those monitoring mortality.

## Editor's Summary

**Background:**

The ability of bacteria to cause infection and disease (that is, their virulence) is in part determined by bacterial genetic makeup. At any time, there is a great deal of genetic diversity within common kinds of bacteria, which naturally generate new variants all the time. Sometimes organisms arise with a genetic makeup that causes more severe infection in humans and even death. For example, in 2005, spread of a particularly virulent strain (called 027/ST1) of the toxin-producing bacterium *Clostridium difficile* caused a global epidemic of *C. difficile* infection.

**Why Was This Study Done?:**

In health care settings, general methods of detecting changing virulence to enable the early recognition, control, and optimal management of increasingly severe infections would be highly beneficial. Changing virulence of a bacterial infection is often measured using death rates, but only a small proportion of those with infection die from it, so this may not be the most sensitive method of monitoring. Consequently, in this study the researchers investigated whether the changing virulence of *C. difficile* infection could be tracked by using common clinical measurements (white blood cell count, neutrophil count, blood urea, and creatinine concentrations, which reflect how unwell patients are) as the basis of an infection-severity surveillance scheme.

**What Did the Researchers Do and Find?:**

The researchers examined all *C. difficile* toxin tests obtained from the microbiological lab serving hospitals in Oxford, UK, between February 1, 1998 and August 1, 2009. They also identified all inpatients aged over 18 years with a positive *C. difficile* test when admitted to hospitals over this time, examined their laboratory blood tests, and noted recorded deaths. The researchers used patients with *C. difficile* toxin-negative samples as a control group. To further validate their analysis, the researchers also undertook a similar analysis in a hospital in another UK city (Birmingham).

The researchers used statistical models to estimate changes in potential biomarkers (neutrophils, creatinine, and urea) with reference to the *C. difficile* 2005 global epidemic and post-infection death rates. The researchers used another statistical model (an iterative sequential regression technique) to estimate how soon any changes in biomarker/mortality trends would have been detected. Finally, to evaluate these severity-monitoring techniques, the researchers performed two simulation studies in which they assumed a more severe strain of *C. difficile* was introduced into a hospital.

Using these methods, the researchers found that in patients who were positive for *C. difficile* toxin, average neutrophil counts on diagnosis increased from 2003, peaked in 2006–2007, and then declined. They also found that 28-day deaths from *C. difficile* infection increased from early 2006, peaked in late 2006–2007, and then declined. Furthermore, laboratory tests (molecular typing) confirmed these changes were likely due to the severe *C. difficile* strain. The simulation model derived from the observed data suggested the performance of biomarker-based detection was notably higher than that of monitoring deaths post-infection.

**What Do These Findings Mean?:**

These findings suggest that passively monitoring the severity of infection using routinely measured clinical biomarkers is feasible and can potentially detect important shifts in the virulence of human pathogens, such as *C. difficile*. Furthermore, such a surveillance system is superior to, and has obvious advantages over, monitoring deaths from infection — provided biomarker data is available. It could be used to provide an early trigger for more detailed investigations of patient characteristics, complemented by studies of bacterial genetic makeup, with the aim of tailoring policy and optimizing treatment of infection. Although this study monitored only one bacterial species, as changes in virulence are common to most human pathogens, this surveillance of severity technique could be applied to other organisms.

**Additional Information:**

Please access these Web sites via the online version of this summary at http://dx.doi.org/10.1371/journal.pmed.1001279.

- Wikipedia provides information about bacterial virulence (note that Wikipedia is a free online encyclopedia that anyone can edit; available in several languages)
- The World Health Organization provides the latest news on infection outbreaks and epidemics
- The Centers for Disease Control and Prevention provide information on *C. difficile* for health professionals and patients
- Trends in *C. difficile* disease in England and Wales are reported by the Health Protection Agency

## Introduction

Bacterial diseases remain one of the most important causes of illness in humans. The genomes, and consequently the clinical behaviours, of many important human pathogens are constantly changing [1]–[4]. Although much previous work has focused on evolving antimicrobial resistance, evolving virulence in successful microbes is also well recognised [5]. One recent example is the global outbreak of ribotype 027/NAP1/ST1 *C. difficile* [6]–[9], in which derepressed toxin production led to enhanced disease severity relative to non-027 strains.

Indeed, continuing genotypic and phenotypic change is probably the rule, rather than the exception, within bacterial species [10]. General methods of detecting changing virulence that would permit early recognition, control, and optimal management of such threats would therefore be highly desirable.

Recent investments in information technology systems in the UK National Health Service, and other large health care providers worldwide, have allowed electronic collection of large amounts of patient data. Such collections typically include various laboratory measurements, in addition to hospital admission and diagnosis data. Changes in parameters, such as peripheral blood white cell and neutrophil counts, urea and creatinine concentrations, can be consequences of an innate response to infection present in all humans [11]. Such changes are known to reflect illness severity in many infections [12]–[15], and form part of generally applicable critical illness scoring systems including APACHE [16].

Since full blood count and renal function measurements are, at least in high-income countries, measured frequently on diagnosis [17], we investigated whether they might form the basis of an infection severity surveillance scheme. We illustrate our approach with an investigation of the changing virulence of *C. difficile* infection (CDI), using data from Oxford Radcliffe Hospitals NHS Trust (ORH), UK.

## Methods

### Data from Oxford and Ethics Statement

We considered all positive *C. difficile* toxin tests obtained in the ORH microbiology laboratory, excluding positive tests within 28 d of a previous positive as a duplicate, according to UK Department of Health guidance [18]. We then identified all inpatients over 18 y of age, with a positive *C. difficile* test whilst admitted to the ORH NHS Trust between 1 February 1998 and 1 August 2009, their associated demographics and hospital admissions prior to the admission with *C. difficile*. The 1998 start date was based on availability of admission data, and the 2009 end date was the time at which the creatinine analysis method used in the hospital changed.

For haematology and biochemical data, we identified the laboratory blood test results closest to the *C. difficile* test sample, within a window extending from 48 h before to 48 h after sampling.

Prior to 1 October 1999, cell-culture-based toxin testing was performed; subsequently, testing used an A+B toxin enzyme immunoassay (Meridian Biosciences). In response to the recognition of an increase in severity in *C. difficile*, from October 2006 onwards, *C. difficile* culture was attempted on all faecal samples with positive toxin tests, *C difficile* isolates stored, and results of multilocus sequence typing [19] were available for toxin-positive culture-positive samples [20],[21]. From May 2007, a change in government policy [18] specified mandatory testing of all unformed stool samples submitted from patients over 65 y, irrespective of perceived clinical risk (whether the patient met local criteria for diarrhoea of ≥3 unformed stools in 24 h).

We excluded patients admitted to oncological and renal specialities, since their laboratory values are likely to reflect their underlying diagnosis, rather than the impact of *C. difficile*, and, for similar reasons, patients with creatinine <16 or >800 µmol/l, neutrophil counts <1×10^9^/l (neutropenia) or >40×10^9^/l, and lymphocyte counts >10×10^9^/l.

All remaining cases were analysed for changes in mortality and incidence over calendar time. Biomarker analyses were restricted to cases with the relevant blood test results. To decrease influence of outliers, for biomarkers with no prior count restriction, values below the 1st percentile and above the 99th percentile were excluded from the analysis.

We considered patients with only toxin-negative samples as a control group. We excluded patients with any subsequent positive tests to minimise the impact of false negatives. A 28-d deduplication was also made for negative tests, and the same inclusion/exclusion criteria were applied.

The data were extracted from an anonymised linked electronic research database, the Infection in Oxfordshire Research Database (IORD), approved by the Oxford Research Ethics Committee (09/H0606/85) and the National Information Governance Board (5-07(a)/2009).

A STROBE checklist is attached as Text S1.

### Data from Birmingham and Ethics Statement

Anonymised microbial isolation and biomarker data were extracted from information systems in the University Hospitals Birmingham NHS Foundation Trust (UHB), in order to assess infection severity in the Queen Elizabeth Hospital and Selly Oak Hospital, Birmingham, at the end of this investigation. UHB is a major tertiary referral centre, organisationally distinct from the Oxford centre, being located about 100 km to the north. These hospitals operate a wide range of medical and surgical services, but not obstetric or paediatric services. As in Oxford, toxin-based testing operated as the diagnostic method throughout the period observed. Data were available from 1 January 2000 onwards, and we deduplicated, selected, and excluded biomarker data as for the Oxford dataset; we used the same end date for analysis as for the Oxford cohort analysed.

### Statistical Methods

We used general linear regression models to estimate changes in mean of potential biomarkers of severity over calendar time. Given the known biology of *C. difficile* infection, we initially investigated neutrophils, creatinine, urea, and albumin, with the aim of identifying whether any marker showed similar calendar trends to those in post-infection mortality and/or indicated the known ribotype 027/ST1 epidemic, which was identified during 2005. Biomarker data were transformed to achieve normality using the Box-Cox method [22], and modelled using normal linear regression; 28- and 7-d mortality were modelled using logistic regression. A gridsearch algorithm, essentially as described [23], was used to identify changes in trends (such as apparent increases or decreases), considering all possible 0- to 4-joinpoint models with joinpoints fitted every 3 mo. The best fitting model was chosen on the basis of the Bayesian Information Criterion (BIC) [24], and models with BIC within 3.84 of the best fit [23] were considered statistically indistinguishable.

To estimate how soon any changes in biomarker/mortality trends would have been detected, we used an iterative sequential regression (ISR) technique. Specifically, we reconstructed what would have been observed if the data had been monitored in real-time, and applied gridsearch to sets of samples taken between 1 February 1998 and 1 September 1998, and successively every month through 1 August 2009. On each dataset a one-trend and a succession of two-trend models were fitted, with iterative selection of optimal joinpoints (see Text S2). Admission neutrophil counts (Box-Cox transformed) in the 027/NAP1/ST1 epidemic clone versus other *C. difficile* strains were compared using *t* tests.

To evaluate the severity monitoring techniques, we performed two simulation studies in which we assumed a more severe strain of *C. difficile* (analogous to ribotype 027/NAP1/ST1) was introduced into a hospital.

The studies assessed the influence of key parameters (additional virulence of the new strain compared to underlying variability in biomarker measurements, penetrance of the new strain, number of affected patients per year) on the following outcome measures: (a) With what probability is the arrival of the severe strain detected? This key operational question was addressed using the ISR method. Data were simulated on the basis of the assumption that after 1 y of constant virulence, a new, more severe, strain was introduced, and during year 2 it replaced existing strains, fully or partially, at a constant rate. The change was deemed ‘successfully detected’ if the best model suggested that the rates were increasing significantly (*p*<0.05) at the study end. (b) If the severe strain arrives, peaks, and then declines (as occurs with successful control), with what probability is control of the outbreak observed? We assumed that during year 1, a new strain arrived and replaced existing ones, as above, and during year 2 it decreased at the same rate. This question is predominantly of retrospective interest; data were analysed using the gridsearch method applied to 0- and 1-joinpoint models. The change was deemed ‘successfully detected’ if the best model contained one joinpoint, located within 6 mo from the real one, and rates decreased significantly (*p*<0.05) at the joinpoint.

Simulation model parameters were derived from the study data (see Text S2 for details). The R language (2.11 for Windows) was used for data visualisation (ggplot2 package), modelling (glm and survival package), and simulation. The source code used in the study can be obtained from http://www.infectionsurveillance.org.

## Results

### Patients Studied

After 28-d deduplication, there were 8,357 *C. difficile* toxin-positive samples from 7,272 adults tested at the ORH NHS Trust between February 1998 through July 2009. We excluded 2,152 cases that were out of hospital and three cases from community hospitals leaving 6,202 samples, of which further 534 cases from renal/oncology specialties and 117 cases with extreme neutrophil, creatinine, and lymphocytes levels were removed (see Methods), leaving 5,551 cases for mortality and incidence analyses. A further 74 cases with extreme urea levels were removed from urea analyses. As a control group, to address possible secular trends in laboratory methods or type of patients tested for *C. difficile*, we used 20,098 deduplicated, and similarly filtered, toxin-negative samples from 17,376 patients who never had a positive test. Demographic details of the *C. difficile* cases and negative controls are presented in Table 1.

**Table 1:** Demographics of the patients studied.

Patient Group	Factor	Subcategory	*n* (%) or Median IQR				
			01/02/1998–2000	2001–2003	2004–2006	2007–01/08/2009	Total
*C. difficile–*positive cases							
	Total	*n* cases	1,043 (100%)	1,638 (100%)	1,790 (100%)	1,080 (100%)	5,551 (100%)
	Sex	Male	440 (42%)	730 (44%)	811 (45%)	463 (42%)	2,444 (44%)
	Age at admission	y	76 (71–86)	76 (71–86)	77 (72–86)	74 (67–85)	76 (71–86)
	Previous ORH admission	In the past year	571 (54%)	907 (55%)	1,158 (64%)	720 (66%)	3,356 (60%)
	Admission specialty codes	General medicine	581 (55%)	985 (60%)	1,110 (62%)	624 (57%)	3,300 (59%)
		Other medicine	226 (21%)	268 (16%)	186 (10%)	90 (8%)	770 (13%)
		General surgery	65 (6%)	134 (8%)	152 (8%)	127 (11%)	478 (8%)
		Trauma and orthopaedic	33 (3%)	63 (3%)	54 (3%)	40 (3%)	190 (3%)
		Other specialties	138 (13%)	188 (11%)	288 (16%)	199 (18%)	813 (14%)
	Missing data	Neutrophil count	330 (31%)	578 (35%)	393 (21%)	144 (13%)	1,445 (26%)
		Urea	371 (35%)	621 (37%)	431 (24%)	176 (16%)	1,599 (28%)
		Creatinine	278 (26%)	531 (32%)	351 (19%)	138 (12%)	1,290 (23%)
		Albumin	460 (44%)	736 (44%)	630 (35%)	313 (28%)	2,139 (38%)
*C. difficile–*negative controls							
	Total	*n* cases	3,121 (100%)	4,330 (100%)	5,142 (100%)	7,505 (100%)	20,098 (100%)
	Sex	Male	1,339 (42%)	1,933 (44%)	2,256 (43%)	3,388 (45%)	8,916 (44%)
	Age at admission	y	68 (57–83)	69 (58–82)	69 (58–83)	70 (60–84)	69 (58–83)
	Previous ORH admission	In the past year	1,417 (45%)	2016 (46%)	2,675 (52%)	3,934 (52%)	10,042 (49%)
	Admission specialty	General medicine	1,420 (45%)	2,043 (47%)	2,529 (49%)	3,556 (47%)	9,548 (47%)
		Other medicine	664 (21%)	709 (16%)	687 (13%)	797 (10%)	2,857 (14%)
		General surgery	374 (11%)	616 (14%)	757 (14%)	1,049 (13%)	2,796 (13%)
		Trauma and orthopaedic	117 (3%)	163 (3%)	168 (3%)	339 (4%)	787 (3%)
		Other specialties	546 (17%)	799 (18%)	1,001 (19%)	1,764 (23%)	4,110 (20%)
	Missing data	Neutrophil count	922 (29%)	1,274 (29%)	1,024 (19%)	1,182 (15%)	4,402 (21%)
		Urea	1,095 (35%)	1,389 (32%)	1,097 (21%)	1,462 (19%)	5,043 (25%)
		Creatinine	779 (24%)	1,153 (26%)	872 (16%)	1,094 (14%)	3,898 (19%)
		Albumin	1,301 (41%)	1,743 (40%)	1,599 (31%)	2,259 (30%)	6,902 (34%)

Changes in incidence over time were similar to national trends [8], increasing from ∼10/10,000 overnight stays in 1998 to ∼16/10,000 overnight stays at the beginning of 2006, then declining back to ∼8/10,000 overnight stays in July 2009 (numbers of cases are shown in Table 1). As noted in Methods, from 2007 onwards, increased testing occurred in low-risk, over 65-y-old patients as required by government policy; this, however, appeared to have little, if any, effect on case demographics (Table 1, and unpublished data). 38% of cases and 34% controls had missing albumin, compared to ≤28% missing data for other biomarkers, and so albumin was not considered further. We found no association between 28-d mortality and missing biomarker values (*p*>0.4).

### Routinely Collected Data Are Associated with Mortality

**Figure 1:**
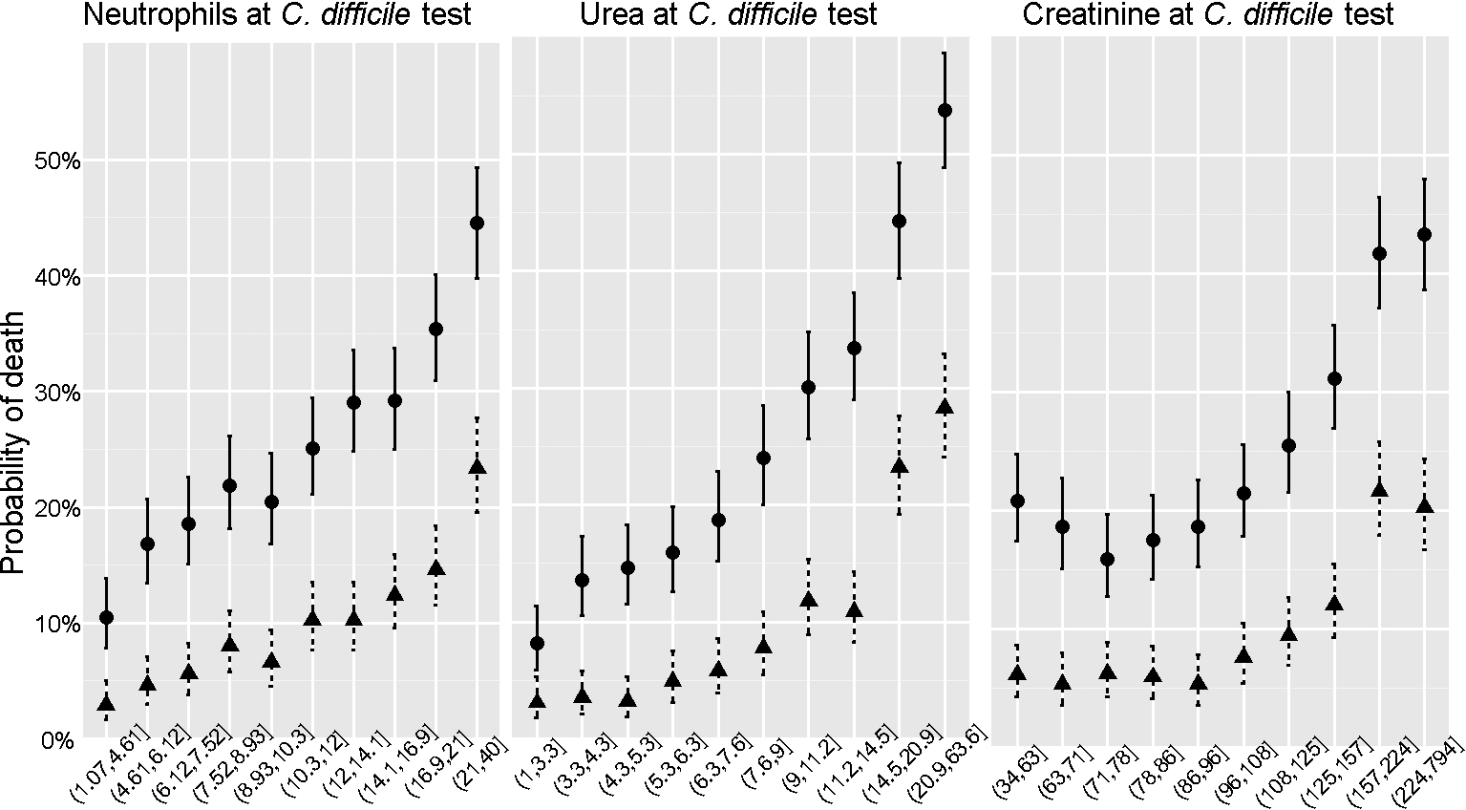
Association between biomarkers and mortality in *C. difficile*-positive patients. The association between mortality within 7 d (triangles) or 28 d (circles) and three biomarkers measured on admission (peripheral blood neutrophil counts×10^9^/l, left, serum urea concentrations (middle, mmol/l), and serum creatinine concentrations (µmol/l, right) are illustrated among the 5,551 cases from the Oxford centre. For each biomarker, patients were divided into deciles of equal size. Each point represents the observed (and 95% CIs around) mortality for patients. Mortality increases as neutrophil and urea concentrations rise. This is also true for creatinine concentrations over about 100 µmol/l.

Elevated levels of neutrophils and creatinine have been reported to be associated with CDI severity and mortality [25]. We confirmed these associations, and an association between raised urea and mortality, in our *C. difficile*–positive cases (Figure 1).

### Secular Changes in *C. difficile-*Associated Severity Markers, Incidence, and Mortality

**Figure 2:**
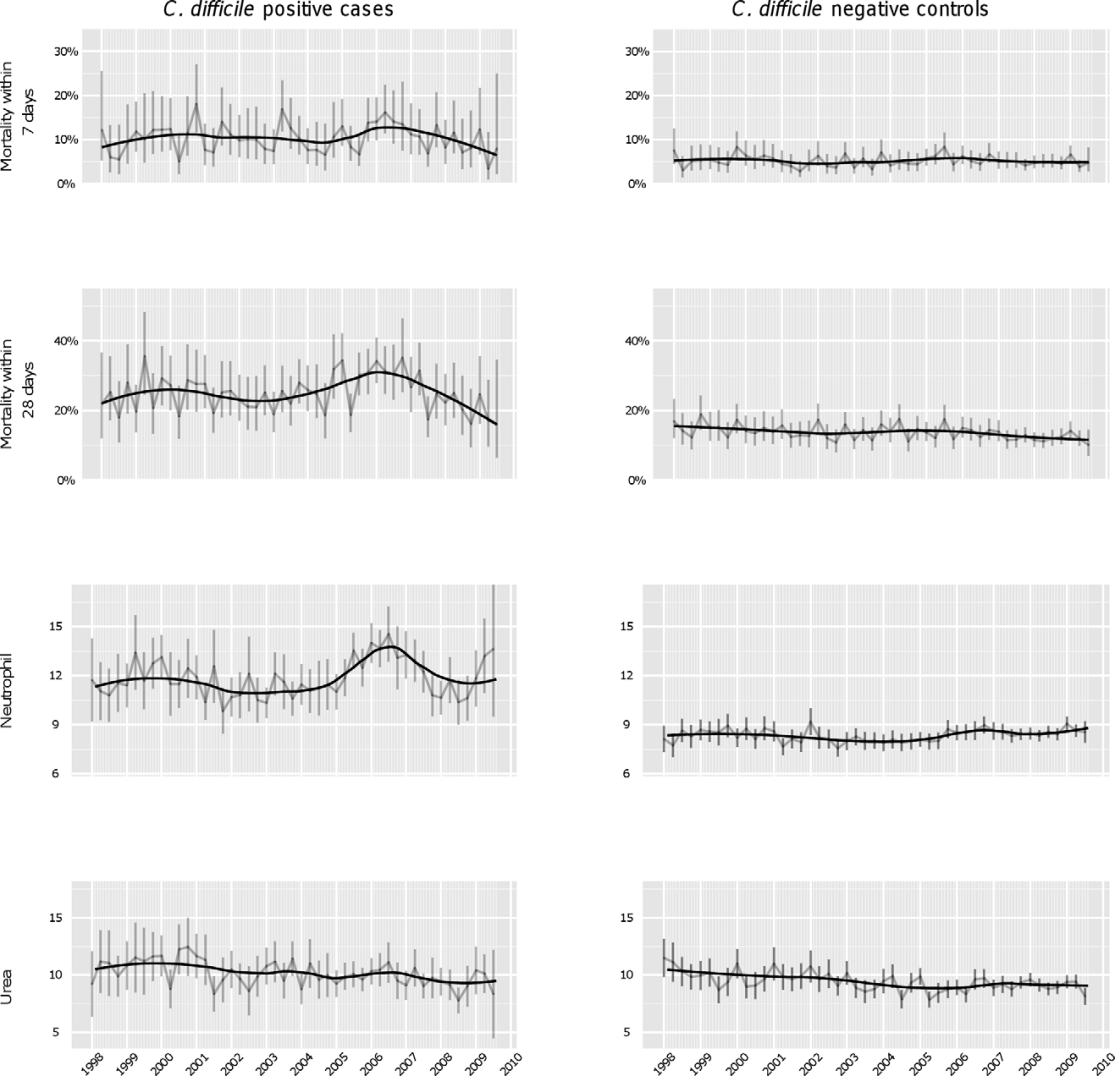
Secular changes in mortality and potential *C. difficile*-associated severity biomarkers. Mean quarterly mortality, neutrophil counts (×10^9^/l), and urea concentrations (mmol/l) between 1998 and 2009 in *C. difficile*-positive cases (left panel) and *C. difficile*-negative controls (right panel) with 95% CIs for each quarter shown as error bars. The black curved lines represent fitted loess curves with the span parameter set to 0.5 to capture the shape of the underlying data. Inspection suggests a rise in neutrophil counts among positive cases between 2004–2009, concomitant with a rise in 28-d mortality. There is also a suggestion of a rise in neutrophil counts among cases presenting in 1999–2000 relative to 2001–2003. Similar changes were not observed in *C. difficile–*negative cases.

Figure 2 shows secular trends in quarterly 7- and 28-d post-infection mortality, and mean neutrophils and urea at CDI diagnosis, over 1998–2009. Notably, persistent elevation in mean neutrophil count at *C. difficile* test between 2005 and 2007 in the *C. difficile–*positive cases is suggested both visually and by loess fitting; trends in mortality were much less apparent, although possibly greater in 7- than 28-d mortality. No equivalent trends were evident in the negative control group for any outcome. There were no obvious trends in urea or creatinine (unpublished data) at CDI diagnosis.

### Retrospective Data Analysis Using Gridsearch Method

**Figure 3:**
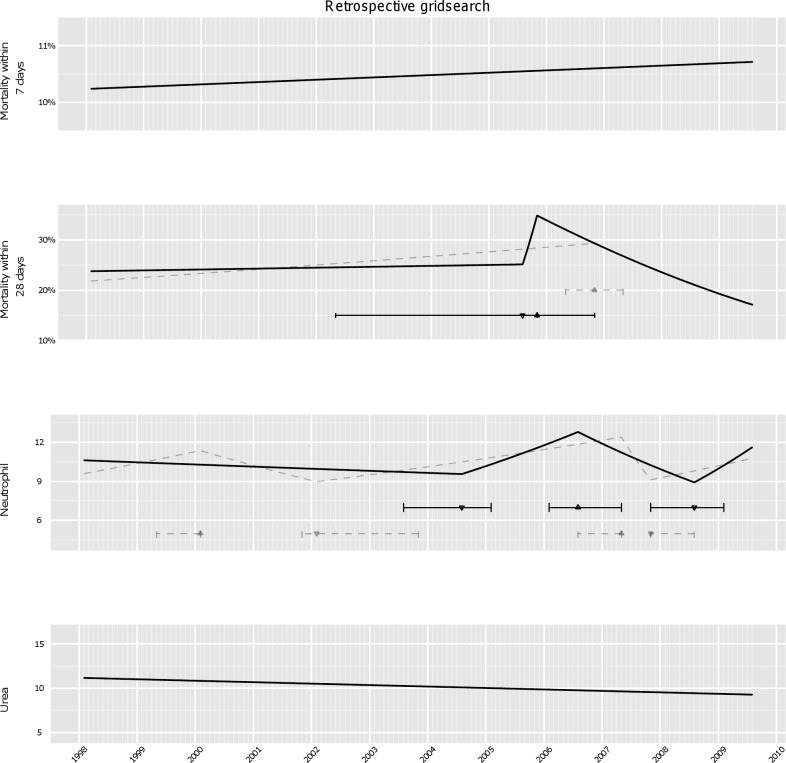
Analysis of secular trends over the whole study period using retrospective gridsearch. The best model for quarterly mortality, neutrophil counts, and urea over the whole study period (solid line) was chosen on the basis of BIC, see Methods. Models with BIC within 3.84 of the best fit were considered statistically equivalent, and variation in the estimated joinpoints across these models are indicated by the horizontal intervals around the joinpoint from the best model (indicating a change from an upward to downward trend by ▴, and from a downward to an upward trend by ▾). Any equivalent models with fewer or greater joinpoints than the best model are shown with dashed grey lines.

The introduction of new strains is often characterised by gradual changes over years [2]–[4]. We therefore used linear models with joinpoints to approximate changes in outcomes over the entire period of the study, as previously done for methicillin-resistant *Staphylococcus aureus* (MRSA) [23]. Up to four joinpoints were permitted (Figure 3).

No trend changes in 7-d mortality were identified: rather 7-d mortality was estimated to have increased slightly but non-significantly over the whole study period (*p* = 0.8). By contrast, the same analyses applied to 28-d mortality detected one single peak, with 28-d mortality increasing sharply from August 2005 through November 2005 and then declining, with statistically indistinguishable models suggesting either that the rise started between May 2002–November 2005, or that 28-d mortality had been increasing gradually but steadily from the start of the study period.

In the best model mean neutrophils started to increase from 2004 (with statistically equivalent models supporting the increase starting between August 2003–February 2005), peaked in 2006 (February 2006–May 2007), and subsequently declined until late 2008 (November 2007–February 2009). Further, some models suggested a weaker earlier rise peaking in 1999 (May 1999–February 2000). Differences between models close (within 3.84 of BIC) to the best model (solid line) were small, with all models identifying a peak in severity in 2006–2007. Similar results were obtained adjusting for age, gender, and previous hospital exposure (unpublished data), suggesting that this biomarker is informative about severity over and above the demographic information. Similar trends were not found in the negative control group (unpublished data), confirming the observed changes are *C. difficile* specific.

The same analysis applied to urea found no evidence of any secular trends, with urea decreasing non-significantly (*p* = 0.16) over the study period, also suggesting that changes in neutrophils could be strain-specific rather than due to other changes in case-mix (Figure 3). Results for creatinine were broadly similar to neutrophils, but with greater intrinsic variability in this biomarker giving less certainty about changes.

### Using ISR Technique for Monitoring Purposes

**Figure 4:**
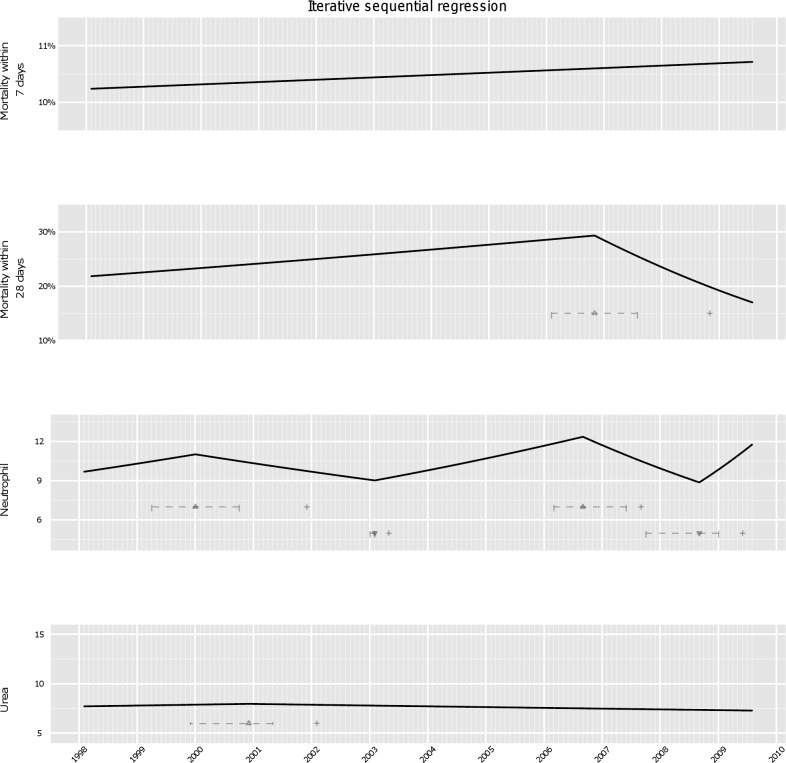
Analysis of secular trends month by month using ISR. Cumulative data on mortality, neutrophils and urea modelled successively to each month, as if such monitoring had been conducted in real-time, see Methods. Changes in secular trends identified by ISR indicated by ▴ (change from an upward to downward trend), and ▾ (a downward to an upward trend), with+indicating the point in time when this change in secular trend was first detected, and horizontal intervals showing the range of joinpoints within 3.84 of the best model BIC at this time.

We used the ISR technique to investigate whether it would have been feasible to use the host response data rather than (or in addition to) post-infection mortality to monitor changes in microbial severity on a regular monthly basis (Figure 4). As across the whole study period, monthly monitoring detected no trend changes at all in 7-d mortality. In contrast, analysis of 28-d mortality identified decreasing mortality after November 2006 only in October 2008. The first time that monthly monitoring would have identified that 28-d mortality was increasing significantly (*p*<0.05) was March 2006.

In contrast, for neutrophil counts we found that an increasing trend from February 1998 changing to a decreasing trend would have been detected in December 2001, with some uncertainty around the exact time of change (April 1999–October 2000), most likely time being January 2000. Interestingly, there was actually an increasing trend in 28-d mortality from February 1998 through January 2000 that did not reach statistical significance (odds ratio [OR] = 1.02 per year, *p* = 0.3).

A new rise in neutrophil levels from February 2003 (January 2003–February 2003) would have been detectable only 3 mo later, in May 2003. Clinically, the ingress of the severe strain into the Oxford hospitals was not detected before national alerts were issued 3 y later and enhanced typing was established in 2006. Neutrophils continued to rise until September 2006 (March 2006–June 2007). The downward change in trend became observable after a year, in September 2007. Intriguingly, the analysis suggests another rise started in late 2008 (*p* = 0.001); molecular analysis of these cases is ongoing. We found no evidence of changes monitoring urea monthly between 2002 and 2009, although there was weak evidence supporting an earlier rise and then decline in 2000 (statistical significance of the change in trend decreased from *p*<0.001 at the detection time to *p* = 0.2 at the end of the study period).

Thus, enhanced severity of *C. difficile* cases peaking around 2006 was strongly suggested by passive monitoring of routine laboratory data using ISR, an efficient joinpoint-based regression method suitable for regular monitoring. Biomarker trends predicted by ISR were broadly similar to those from a much more computationally intensive gridsearch method (Figure 3).

### Non-Parametric Modelling

We investigated whether changes in severity markers might have been more noticeable in only a subset of the population, such as patients with more severe infection, using non-parametric (quantile) regression for the 75th and 90th quantiles. Both gridsearch and ISR techniques detected the 2000 and the 2006 peaks for neutrophil counts. However, in some models multiple additional joinpoints were suggested (unpublished data), as a consequence of more variability in these quantiles.

### Prediction of Enhanced Virulence Confirmed by Molecular Typing

**Figure 5:**
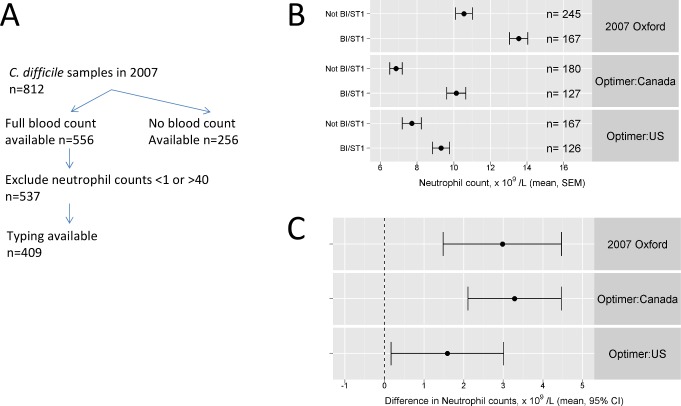
Validation of predictions of passive surveillance: molecular typing data from ORHs and from the 003/004 studies. Flowchart (A) illustrates laboratory and typing data available for consecutive faecal samples with positive *C. difficile* toxin tests in the Oxford site during the 2007 calendar year. Diagnosis peripheral blood neutrophil counts in ST1/NAP1/027 versus non-ST1/NAP1/027 cases are shown for three cohorts: Oxford in 2007, US patients entered into the 003/004 fidaxomicin studies, and Canadian patients entered into the same trials. (B) Means and standard errors in each group are shown, as are (C) 95% CIs around the difference between ST1/NAP1/027 versus non-ST1/NAP1/027 cases.

One explanation for the observations of increased neutrophil counts in those with CDI is that the strain(s) of *C. difficile* prevalent in 2006 exhibited enhanced virulence relative to those in 2003. A multi locus typing scheme (MLST) was established in late 2006 (see Methods). During the first full year of this program (2007), as the neutrophil severity signal was beginning to decline (above), full blood count data and typing information were available on 409 patients with culture confirmed *C. difficile* (Figure 5). Mean neutrophil count among the 167 ST1 cases was 13.5×10^9^/l, while in the 245 non-ST1 isolates it was 10.7×10^9^/l (difference in means 2.8 [95% CI 1.5–4.5], *p* = 0.0001). Quantile regression gave an almost identical estimate (2.8, 95% CI 1.50–4.61). Thus, comprehensive typing in one large group of hospitals suggests the ingress of the virulent 027/NAP1/ST1 C. *difficile* strain is the most likely explanation for the elevation in neutrophil counts. It further suggests the onset of the epidemic of severe disease began in Oxfordshire in around 2003.

### Generalizability of the Technique

To ascertain whether the biomarker-monitoring technique we have described could be generally applicable, we performed three further investigations. Firstly, we determined whether the biomarker∶severe strain association observed in Oxford was present in other settings. Secondly, we performed simulation studies to assess its likely performance in future outbreaks in other settings, relative to the monitoring of mortality. Thirdly, we analysed the trends in severity-associated biomarkers in another hospital using the ISR technique.

### Multi-centre Investigation into the Neutrophil: Strain-Type Association

We examined unpublished associations between admission biomarker and microbial culture data from two pivotal phase III double-blind randomized non-inferiority licensing trials (studies 003 and 004) of oral fidaxomicin versus vancomycin in the treatment of *C. difficile* disease [26],[27]. Recruitment was predominantly from North America: Study 003 recruited from 62 sites in North America, whereas study 004 recruited from 86 sites: 41 from US and Canada, and 45 from Europe.

Eligibility criteria in the 003/004 studies involved ELISA-based toxin testing, rather than culture. Organisms were cultured and typed from a proportion of the patients included, as discussed [26],[27]. PCR ribotyping was used as a typing method [26] rather than MLST, but PCR-ribotype 027 has been shown consistently to be ST1 (and vice versa) [19],[28]. The non-cultured cases did not differ systematically from the culture positive cases (unpublished data).

Of the cases who had both culture results and admission blood counts available, there were 600 North American patients, 293 from the US and 307 from Canada. The European group, which was smaller (*n* = 123, of which 22 were from ORH) was not analysed further because of overlap with the previous study.

Figure 5 shows mean neutrophil count among the cases with the 027 severe strain was significantly higher in both US and Canadian patients than in cases with other strains 9.3×10^9^/l, and 10.1×10^9^/l, respectively, while in the non-027 cases it was 7.7×10^9^/l and 6.8×10^9^/l, respectively (*t* test, *p*<1×10^−4^ and 0.01, respectively). Non-parametric rank-sum testing produced similar conclusions (*p* = 0.002, *p* = 0.01, respectively). Thus, the difference between severe strain biomarkers was found in diverse sites, suggesting severity biomarker monitoring may be widely applicable. The mean counts were somewhat lower in the 003/004 studies than in the Oxford typing series, although the difference between the strains is very similar. This is likely due to both the younger age in the 003/004 studies (mean 62 y) compared to ORH (mean 75 y), and trial eligibility criteria, particularly the need for written informed consent and the exclusion of very severe cases [26],[27].

### Simulation Studies

**Figure 6:**
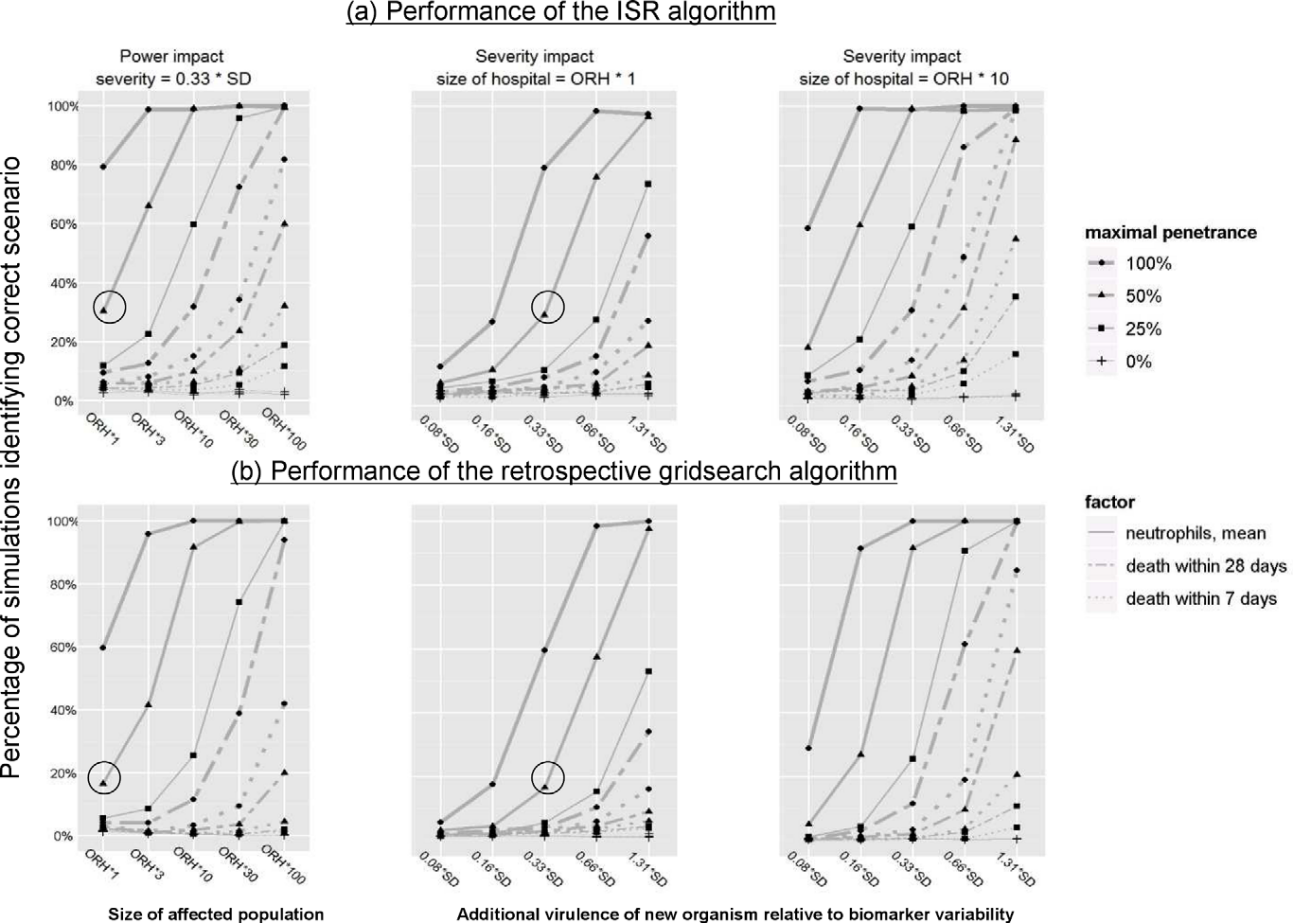
Simulation studies investigating the impact of virulence, penetrance, and study population size on monitoring performance. The study parameters included size of the hospital(s) being monitored (left panel), additional virulence of the new strain relative to biomarker variability (central and right panels); and maximal penetrance of the new strain (all panels, effect represented by lines of different width). The simulated scenarios investigated the probability with which the arrival (top panels) or decline (bottom panels) of the new strain could be detected, see Methods. The circle corresponds to the most likely parameters of the 2006 outbreak.

To further assess the potential of the severity surveillance techniques described, we simulated two scenarios (see Methods): one in which a new, severe organism invades a hospital (Figure 6a), and a second in which it first invades, and then declines, for example due to effective infection control (Figure 6b).

Based on realistic simulation parameters derived from our observed data (see Text S2), the performance of biomarker-based detection was notably higher than that of post-infection mortality monitoring. To illustrate, if the new strain had been four times as virulent as the original 027/NAP1/ST1 strain and increased to 50% of circulating strains, biomarker monitoring would have identified its arrival in 96% cases, as opposed to 20% with mortality monitoring (Figure 6a).

As expected, increased virulence and/or penetration of the new strain results in changes being detected more often, regardless of monitoring method. For example, the arrival of a strain with 2-fold increased virulence would have been detected in ORH in 76% as opposed to 30% cases for the baseline case (Figure 6a). However, in an ORH-sized hospital, a new organism would have needed to have been four times as virulent and have increased to 100% penetrance to have been detected in >50% cases using 28-d mortality monitoring.

Enhanced power could also be achieved using a multi-centre network of hospitals. For example, a 10-fold increase in the hospital-monitored population would have resulted in a 92% detection rate for control of a new organism, in comparison with the 16% detection rate of the baseline case (Figure 6b).

The rate of identifying (false-positive) changes in the simulations with no new strain remained under 5% in both simulations.

### Studies of Trends in Other Hospitals

Finally, we used the ISR technique to analyse trends in neutrophil counts in a second major UK hospital, based in Birmingham. Data were not available prior to 2000 in this centre. Following exclusions identical to those operating in Oxford, there were 5,399 cases available for analysis.

**Figure 7:**
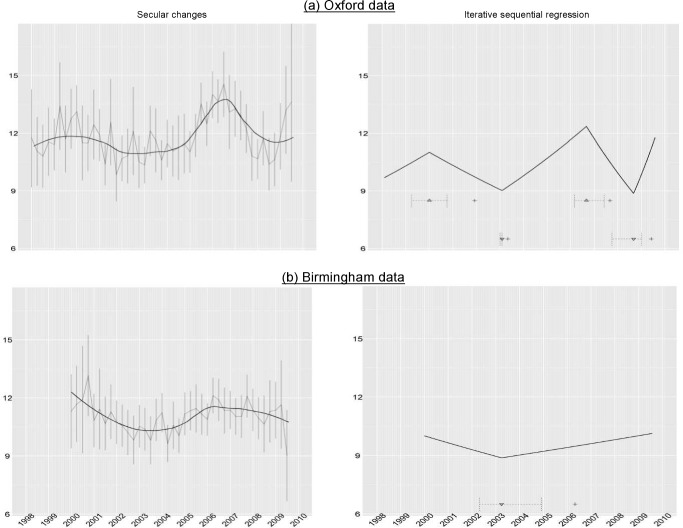
Comparison of secular changes in neutrophils across Oxford (top panels) and Birmingham (bottom panels) hospitals. Left panels contain mean quarterly neutrophil counts, with 95% CIs shown in grey, and black lines representing the loess curves with the span parameter set to 0.5. Right panels show changes in secular trends identified by ISR indicated by ▴ (change from an upward to downward trend), and ▾ (a downward to an upward trend), with+indicating the point in time when this change in secular trend was first detected, and horizontal intervals showing the range of joinpoints within 3.84 of the best model BIC at this time.

Data are presented over the same time frame as used for the Oxford study (Figure 7).

Interestingly, a decline in neutrophil counts on presentation was seen in the Birmingham centre from 2000–2003, as was observed in Oxford, compatible with a period of increased severity of *C. difficile* in the UK around 2000. Similar to the Oxford centre, an increase in severity was observed from 2003 onwards, and which was detected by the ISR algorithm, although with some uncertainty about the change point. Visual inspection suggests that, as in Oxford, neutrophil counts declined after a peak in or around 2007 to 2008. The change downwards is not detected by ISR in the data shown in Figure 7, although it was detected in subsequent data (unpublished data).

In summary, similar trends in severity are observed in two UK hospitals, and are detected by the ISR algorithm.

## Discussion

In this study of infection severity surveillance, we used *C. difficile* disease as an example, because enhanced virulence associated with emergence of a new strain (027/NAP1/ST1) was known to have occurred historically. Widespread clinical recognition of clone impact, including in Oxford Radcliffe Hospitals, occurred in 2006; retrospectively, the clone was detected in stored samples across Europe from 2003 onwards [8],[9], the same time that passive monitoring of neutrophils would have suggested a significant increase in infection severity in our hospitals. This evidence that the 027/NAP1/ST1 strain caused the severe disease observed is supported by experimental studies in animals [7],[27],[29].

For evaluation of our passive surveillance technology, we mainly focused on neutrophil counts on diagnosis, because of their frequent measurement prior to therapy, and their known association with mortality in *C. difficile* colitis [8], something also seen in our study. We elected not to study measures that could be influenced by secular trends in either clinical practice or therapeutic efficacy, such as duration of stay or speed of resolution of host response.

Visual inspection, gridsearch, and ISR analyses of passively collected laboratory measures of severity (neutrophil counts and, to a lesser extent, creatinine) were all highly suggestive of rising pathogen virulence from 2003–2004 to 2006, followed by virulence decline. The prediction that these changes were due to ingress of the highly virulent ribotype 027/NAP1/ST1 strain is supported by detailed molecular typing data in one centre. Further, such severe strain-associated biomarker associations were also found in two clinical trials performed across multiple hospitals, and are compatible with published literature from diverse sites [6]–[8],[30]. Similar secular trends in biomarkers were observed in another large hospital group. Together, these data support the generalizability of the initial observations from a single large hospital group.

The likely utility of the biomarker-monitoring technique described here has been assessed by simulation. Importantly, analysis of both the historical outbreak, and of future simulated outbreaks, shows monitoring of post-infection mortality, as opposed to laboratory measures of severity on diagnosis, affords much less confident detection. This probably reflects the relatively low attributable mortality of *C. difficile* [31], and the lesser statistical power inherent in use of a dichotomous, rather than continuous, variable.

Interestingly, in addition to the known 2006 outbreak, regression models suggest another rise of neutrophil counts peaking around 2000, which was seen in two hospitals. This suggests that waves of virulence may be a recurring feature of *C. difficile*, something compatible with observed ongoing heterogeneity of virulence in the *C. difficile* population [32]. However, the basis of the earlier severity peak remains speculative, as typing data are not available from 2000.

### Generalizability

To what extent would one expect this kind of biomarker-based surveillance to be generalizable to other organisms and conditions? It requires that there exists at least one routinely collected biomarker that is associated with disease-related mortality for each target condition. This is likely to be true for many infections; measures of renal function (urea, creatinine), glucose levels, platelet counts, measures of deranged liver function (transaminases, etc.), and coagulation status have all been shown to be prognostic in various infections [12]–[16],[33]. Indeed, such biomarkers are well recognised in studies of all-cause critical illness cohorts, further supporting the likely generalizability of the technique [16]. In practice, we envisage that initially a number of potential severity markers could be investigated for each infection, retrospectively using historical data if available, or prospectively based on routine electronic databases. Comparing historical data with mortality retrospectively, and/or investigating any “signals” prospectively, would identify which biomarkers were most useful for passive severity monitoring.

With all surveillance systems, the populations monitored need to be carefully defined. Obviously, choosing to run analyses on poorly defined subgroups (or outcomes) may result in ‘effect dilution’ if severity change only occurs in part of the population studied; our simulation studies, presented here, will allow readers to judge the likely impact of such effect dilution in their populations.

### Limitations

Routine monitoring of laboratory measures of severity has limitations. One concerns feasibility. The samples used to predict severity were routinely collected, and came from inpatients; although in many hospitals in high-income countries such samples are taken in the majority of admissions, this may not be the case in less resourced settings.

A less obvious issue is biological, and concerns the constancy of the biomarker∶mortality relationship. Inter-human variation is a contributor to the noise in this relationship [26], but the surveillance technique described here can operate successfully despite this, given adequate sample numbers. It is possible that harmless strains of common microbes might exist that enhance biomarkers but do not enhance mortality, or perhaps vice versa. In practice, innate immune microbial recognition, which drives many of the biomarkers whose changes we suggest could be monitored, is multi-faceted [6], and strong selective pressures have operated over millennia to consistently detect and respond in a protective manner to all pathogens [6]. Consequently, we suspect differential innate immune stimulation by different microbial strains will be limited, although clearly such variant(s) may exist, and remain to be discovered. In particular, we are not aware of published work describing heterogeneity in the host response: mortality relationship within members of a bacterial species. However, even if this heterogeneity were to exist, a biomarker that was even a partial surrogate for mortality could still provide substantial additional power to detect severity changes over current methods that rely on clinician suspicion or mortality monitoring.

What action should be taken on the basis of ‘signals’ from this kind of surveillance? We suggest, on the basis of the experience with *C. difficile*, one should not be particularly reassured if anecdotal clinical opinion suggests there is ‘no obvious problem’, while severity surveillance is identifying rising biomarker levels, as heterogeneity of severity, low frequency of clinician contact with particular infections, and the insidious nature of severity changes may all militate against clinical detection. Rather, we would advocate further analytical investigation(s). The requirement for such additional study is a feature of much passive surveillance and applies equally to more widely used techniques for monitoring incidence and mortality; the investigation could be either prospective or retrospective, perhaps across hospitals, and would aim to identify whether patient characteristics, or strain characteristics, were associated with the observed severity signal. In some situations where microbiological diagnosis is rare, such as pneumonia, it might involve enhanced sample collection. In such investigations, modern molecular typing methods, which have very high sensitivity for the detection of new strains [30], but also identify much variation that is unrelated to severity, would likely complement the sort of routine biomarker-based surveillance we describe here, which is capable of monitoring severity changes in organisms that are not routinely cultured, such as *C. difficile*.

There are also statistical issues related to the technique used in this paper, which is not necessarily optimal: simultaneous use of multiple measures of severity (such as neutrophil and creatinine counts together), perhaps using Bayesian techniques, may offer increased sensitivity and more rapid detection, without increase in false positive results. Detailed comparison with existing changepoint detection algorithms [34]–[38] is planned. Preliminary results suggest that at least in some scenarios, such as those described in the simulation study, ISR performance is superior to that of the widely used scan-statistic based tool, SaTScan (unpublished data) [39]. In contrast, SaTScan is superior for detecting highly localised ‘outbreaks’ [40], where changes are rapid and short-lived as opposed to the more gradual changes that typically accompany strain variation.

Independent of the optimal algorithm, however, we have shown that monitoring severity of infection passively is feasible, can detect important shifts in the phenotype of human pathogens, and offers superior performance relative to mortality monitoring. Since waves of virulence, as shown here with *C. difficile*, appear to be a general feature of the evolution of human microbial pathogens [1]–[4],[10], we believe the approach described here, which we term ‘surveillance of severity’, will be applicable to many organisms and syndromes.

## Supporting Information

Text S1 **STROBE checklist.**
(DOC)

Text S2 **Statistical appendix.**
(DOC)

## Abbreviations

BIC: Bayesian Information Criterion
CDI: *C. difficile* infection
ISR: iterative sequential regression
ORH: Oxford Radcliffe Hospitals
