# Supplementary material for: Surveillance of Infection Severity: A Registry Study of Laboratory Diagnosed Clostridium difficile
Source: PLoS Med. 2012 Jul 31;9(7):e1001279. doi: 10.1371/journal.pmed.1001279 (PMC3409138; doi:10.1371/journal.pmed.1001279)
Supplement: Text S1 — STROBE checklist. (DOC) [file pmed.1001279.s001.doc]

STROBE Statement—Checklist of items that should be included in reports of ***cohort studies***

|  | Item No | Recommendation |
| --- | --- | --- |
| **Title and abstract** | 1 | (*a*) Indicate the study’s design with a commonly used term in the title or the abstract  See Title |
|  |  | (*b*) Provide in the abstract an informative and balanced summary of what was done and what was found  See Abstract |
| Introduction | | |
| Background/rationale | 2 | Explain the scientific background and rationale for the investigation being reported  Introduction PARA 1,2 |
| Objectives | 3 | State specific objectives, including any prespecified hypotheses  Introduction PARA 3,4 |
| Methods | | |
| Study design | 4 | Present key elements of study design early in the paper  Methods para 1-4 |
| Setting | 5 | Describe the setting, locations, and relevant dates, including periods of recruitment, exposure, follow-up, and data collection  See methods sections ‘Data from …’ |
| Participants | 6 | (*a*) Give the eligibility criteria, and the sources and methods of selection of participants. Describe methods of follow-up  See notes under ‘Setting’. |
|  |  | (*b*) For matched studies, give matching criteria and number of exposed and unexposed  N/A |
| Variables | 7 | Clearly define all outcomes, exposures, predictors, potential confounders, and effect modifiers. Give diagnostic criteria, if applicable  See notes under ‘Setting’ |
| Data sources/ measurement | 8* | For each variable of interest, give sources of data and details of methods of assessment (measurement). Describe comparability of assessment methods if there is more than one group  See notes under ‘Setting’ |
| Bias | 9 | Describe any efforts to address potential sources of bias  See notes under ‘Setting’ including sections on replication in multiple centres, exclusion criteria and justification. |
| Study size | 10 | Explain how the study size was arrived at  See methods; see also details of simulation (PAGE 5) for discussion of power |
| Quantitative variables | 11 | Explain how quantitative variables were handled in the analyses. If applicable, describe which groupings were chosen and why  See Methods. |
| Statistical methods | 12 | (*a*) Describe all statistical methods, including those used to control for confounding  See section in Methods, & Statistical appendix |
|  |  | (*b*) Describe any methods used to examine subgroups and interactions  N/A |
|  |  | (*c*) Explain how missing data were addressed  EXCLUSION; see Methods. |
|  |  | (*d*) If applicable, explain how loss to follow-up was addressed  N/A |
|  |  | (*e*) Describe any sensitivity analyses  See methods & statistical appendix. |
| Results | | |
| Participants | 13* | (a) Report numbers of individuals at each stage of study—eg numbers potentially eligible, examined for eligibility, confirmed eligible, included in the study, completing follow-up, and analysed  Results para 1. |
|  |  | (b) Give reasons for non-participation at each stage  Results para 1. |
|  |  | (c) Consider use of a flow diagram  See results We did produce a flow diagram, but have not included it as all relevant numbers are in Results para 1. |
| Descriptive data | 14* | (a) Give characteristics of study participants (eg demographic, clinical, social) and information on exposures and potential confounders  Table 1 |
|  |  | (b) Indicate number of participants with missing data for each variable of interest  Table 1 |
|  |  | (c) Summarise follow-up time (eg, average and total amount)  7 & 28 day mortality available in all cases, see initial sections of results. |
| Outcome data | 15* | Report numbers of outcome events or summary measures over time  Table 1, Figure 1,2 |
| Main results | 16 | (*a*) Give unadjusted estimates and, if applicable, confounder-adjusted estimates and their precision (eg, 95% confidence interval). Make clear which confounders were adjusted for and why they were included  See Results for discussion of adjustment for confounding. |
|  |  | (*b*) Report category boundaries when continuous variables were categorized  N/A |
|  |  | (*c*) If relevant, consider translating estimates of relative risk into absolute risk for a meaningful time period  N/A |
| Other analyses | 17 | Report other analyses done—eg analyses of subgroups and interactions, and sensitivity analyses  See results, including multi-centre studies. |
| Discussion | | |
| Key results | 18 | Summarise key results with reference to study objectives  P11 para 1-3 |
| Limitations | 19 | Discuss limitations of the study, taking into account sources of potential bias or imprecision. Discuss both direction and magnitude of any potential bias  See para 4-9 |
| Interpretation | 20 | Give a cautious overall interpretation of results considering objectives, limitations, multiplicity of analyses, results from similar studies, and other relevant evidence  Done, paras 4-9. |
| Generalisability | 21 | Discuss the generalisability (external validity) of the study results  Discussed in context of multi-centre replication. |
| Other information | | |
| Funding | 22 | Give the source of funding and the role of the funders for the present study and, if applicable, for the original study on which the present article is based  Done, see funding section. |

*Give information separately for exposed and unexposed groups.

**Note:** An Explanation and Elaboration article discusses each checklist item and gives methodological background and published examples of transparent reporting. The STROBE checklist is best used in conjunction with this article (freely available on the Web sites of PLoS Medicine at http://www.plosmedicine.org/, Annals of Internal Medicine at http://www.annals.org/, and Epidemiology at http://www.epidem.com/). Information on the STROBE Initiative is available at http://www.strobe-statement.org.
