# Supplementary material for: Surveillance of Infection Severity: A Registry Study of Laboratory Diagnosed Clostridium difficile
Source: PLoS Med. 2012 Jul 31;9(7):e1001279. doi: 10.1371/journal.pmed.1001279 (PMC3409138; doi:10.1371/journal.pmed.1001279)
Supplement: Text S2 — Statistical appendix. (DOC) [file pmed.1001279.s002.doc]

## Statistical Appendix

Accessing source code: code is available from http://www.infectionsurveillance.org

## Assumptions in simulation studies

In the base simulation we assumed that

- there were 365 affected patients per year (approximate ORH burden in 2008-2009);
- the neutrophil counts, transformed with the Box-Cox function *y=(x^λ^ – 1)/λ* with λ=0.2, were normally distributed with standard deviation (SD) of 0.93, means of 2.95 and 3.26 for non-ST1 and ST1 strains respectively (ie additional virulence associated with ST1 is 0.33*SD), truncating at 0 and 5.46, the transformed value corresponding to the exclusion criteria of >40x10^9^/l and <1x10^9^/l in our study
- maximal ST1 penetration of 50%
- the probability of survival followed the Weibull distribution

$\log(p(t))=-e^{-\left(\frac{\text{intercept}+\text{neutrophils}+\text{ST1/non-ST1}}{scale}\right)}*t^{\frac{1}{scale}}$

with coefficients estimated from our observed data, namely intercept=6.84, (transformed) neutrophils (-0.51 per 10^9^/l ), effect of ST1 vs non-ST1 strain (‑0.42) and scale 1.26.

The simulation studies assessed the influence of the following parameters:

(i) additional virulence of the new strain compared to underlying variability in biomarker measurements, assumed to be 0.08xSD, 0.16xSD, 0.33xSD (base case), 0.66xSD and 1.31xSD,

(ii) penetrance of the new strain, assumed to be 0% (null hypothesis), 25%, 50% (base case) and 100%,

(iii) number of affected patients per year, assumed to be 365 (ORH*1, base case), ORH*3, ORH*10, ORH*30 and ORH*100.

Each simulated scenario was repeated 1000 times.

## ISR details and algorithm

On each dataset a one-trend and a succession of two-trend models were initially fitted. When the one-trend model had lowest BIC (or BIC within 3.84 of the best two-trend model), the endpoint was moved forward by a month and the algorithm repeated. Whenever a two-trend model had a significantly better (BIC lower by at least 3.84) than the best one-trend model, the joinpoint corresponding to the best two-trend fit was fixed in the following steps, so that subsequently the algorithm only looked for new joinpoints in the interval between the last fixed joinpoint and the current endpoint. Notably, the precise increase/decrease around the joinpoint change was not fixed, being re-evaluated on each subsequent fit. Similar to above, at each stage, in addition to the fixed joinpoints, the joinpoints corresponding to models with BIC within 3.84 of the best fit were also identified. Finally, based on known likely biological behaviour of emerging infectious threats, we constrained the distance between joinpoints and the distance between a joinpoint and its detection time, to be at least three months. The algorithm for the procedure is shown below.

Initial values:

dataset := records between 01/02/1998 and 01/08/2009

end date t := September 1998

set of joinpoints J := emptyset

indistinguishable set of joinpoints CI(J) := emptyset

time of detection T(J): = emptyset

Iterate over months through to t=August 2009

Set dataset(t) := records in the dataset between 01/02/1998 and t.

Set M:=model with joinpoints J, fitted on dataset(t).

For each month s between max(J)+3 months (May 1998 if J=emptyset) and t-3 months

set M := (M, model with joinpoints (J, s), fitted on dataset(t)).

Consider “best” models only, so set

m := model from M with the lowest BIC;

M := the models in M with BIC within 3.84 of BIC(m).

If the model with joinpoints J is not in M, set

J := (J, joinpoints of m);

CI(J) := (CI(J), joinpoints of all m in M)

T(J) := (T(J), t).

Set t := t + 1 month.

Output:

J: joinpoints corresponding, at each step, to the model with lowest BIC;

CI(J): joinpoints corresponding, at each step, to the models with BIC within 3.84 of each model with lowest BIC;

T(J): joinpoint detection times.
